# Viral load testing in a resource-limited setting: quality control is critical

**DOI:** 10.1186/1758-2652-14-23

**Published:** 2011-05-12

**Authors:** Jane Greig, Philipp du Cros, Derryck Klarkowski, Clair Mills, Steffen Jørgensen, P Richard Harrigan, Daniel P O'Brien

**Affiliations:** 1 Manson Unit, Médecins Sans Frontières, London, UK; 2 Public Health Department, Médecins Sans Frontières, Amsterdam, Holland; 3 Department of Clinical Immunology, Hospital South, Naestved, Denmark; 4 BC Centre for Excellence in HIV/AIDS, Vancouver, BC, Canada

## Abstract

**Background:**

World Health Organization guidelines now recommend routine use of viral load testing, where available, for patients receiving antiretroviral treatment (ART). However, its use has not been routinely implemented in many resource-limited settings due to cost, availability and accessibility. Viral load testing is complex, making its application in resource-limited settings challenging. We describe the issues encountered by Médecins Sans Frontières (MSF) when using routine viral load testing in a large HIV programme in sub-Saharan Africa.

**Methods:**

Between October 2005 and August 2006, more than 1200 patients on ART had viral load tests at baseline and at three-month intervals performed by a local reference laboratory that was quality assured by an experienced international institution. Concerns with reliability of results halted testing. The quality control measures instituted with a second laboratory and outcomes of these were documented.

**Results:**

In 2005 and 2006, only 178 of 334 (53%) previously ART-naïve patients tested after six to 12 months of treatment had viral loads of less than 1000 copies/mL. Similar MSF programmes elsewhere demonstrated virological suppression rates of more than 85%, and duplicate testing showed unacceptable discordance. Laboratory problems encountered included: disregarded quality control; time delays; requirement for retesting; and duplicate sample variations. Potentially harmful clinical outcomes of inaccurate viral load results include: unnecessary ART regimen changes; unnecessary enhanced adherence counselling after "false failures"; and undetected virological failure.

**Conclusions:**

Viral load testing performed without rigorous quality control carries the risk of erroneous and potentially damaging results. Viral load testing should be utilized only if robust quality assurance has been implemented. Our experience in this and other settings led to the development of a guide for assessing the suitability of a laboratory for viral load testing that can be used to help achieve reliable results.

## Background

Viral load (VL) testing is the only definitive method for early detection of antiretroviral treatment (ART) failure [1,2]. HIV treatment can be managed without routine laboratory assessment [3], but CD4 monitoring allows for evaluation of disease progression [4], and VL testing can increase adherence and facilitate timely switching of failing regimens, minimizing the development of resistance [5]. VL testing has not been routinely implemented in resource-limited settings due to cost, complexity, availability and accessibility. Calls for widespread VL test use are increasing, and 2009 World Health Organization (WHO) guidelines recommend the routine use of VL testing where available [6,7]. However, obtaining quality controlled and reproducible results for even simple laboratory tests in resource-limited settings is challenging [8,9]. Inaccurate or delayed results can have serious consequences for patients and programmes.

**Figure 1:**
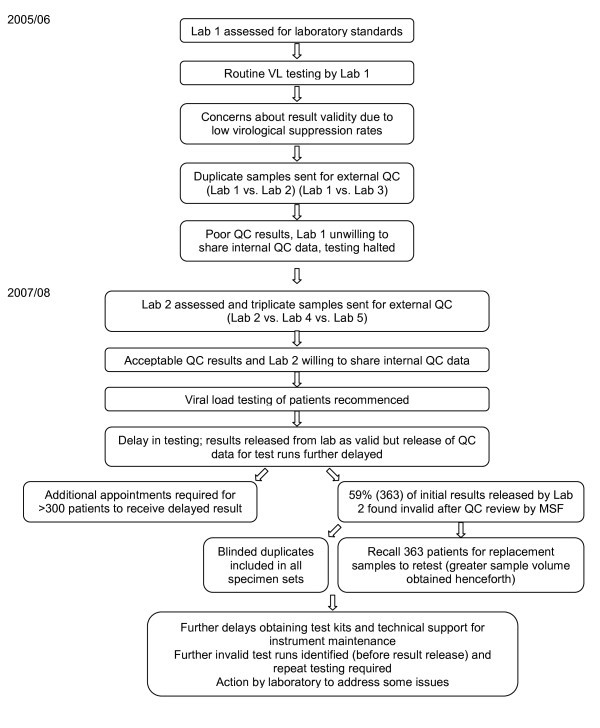
**Flow of events**. VL - Viral load. QC - Quality control

An HIV programme run by Médecins Sans Frontières (MSF) in an urban resource-limited setting in sub-Saharan Africa offered free medical care and psychosocial support to people living with HIV/AIDS. Patients could self-refer for on-site counselling and testing, or were referred by other healthcare providers. Over a period of more than five years, MSF registered more than 2700 adults living with HIV/AIDS and provided ART to almost 2000. Most were women (63%), and many survived on less than US$2 per day. We describe the experience of routine VL testing for patients receiving ART in this HIV programme in two separate series of events between 2005 and 2008 (Figure 1).

## Methods

Between October 2005 and August 2006, more than 1200 patients on ART had VL tests at baseline, and every three months thereafter. Samples were collected in EDTA tubes, centrifuged for 10 minutes at 800 × g and the plasma stored in sterile tubes at -20°C for less than eight weeks. Samples were measured at a local reference laboratory (Lab 1) using Roche Cobas Amplicor HIV-1 Monitor Test v1.5 and Cobas Amplicor Analyser (Roche Diagnostics, Basel, Switzerland), with preparation of frozen plasma in accordance with the Amplicor v1.5 manual guideline.

Prior to use, Lab 1 was assessed by an MSF laboratory scientist for: training of personnel; appropriate laboratory facilities; workflow; separation of areas for sample preparation, reagent preparation and sample analysis; backup power for laboratory freezers and refrigerators; temperature monitoring guidelines; cleaning guidelines for different laboratory work areas; and access to water of appropriate quality. The laboratory was found to be of a high standard, and was supported and quality assured by an international institution with experience in VL testing, with tests performed by trained staff already experienced with the procedures. No external quality control was organized by MSF.

Due to concerns regarding the validity of results, sample collection and preparation procedures were reviewed and reinforced, and additional samples were tested as blinded duplicates: 21 duplicates were sent to Lab 1 and another laboratory in-country (Lab 2, which was internationally supported, experienced in performing VL tests, and had good facilities found during assessment by an MSF laboratory scientist; VL testing was performed using Bayer Versant HIV-1 RNA 3.0 bDNA assay with Bayer System 340 bDNA Analyzer [Bayer Diagnostics, Tarrytown, NY, USA] with samples prepared according to the Versant guidelines). A further 29 duplicates were sent to Lab 1 and a reference laboratory in another sub-Saharan African country (Lab 3) that used Roche Amplicor. The differences between paired log VL results and the mean absolute differences for all samples were calculated, and the limits of agreement between laboratories compared using the method of Bland and Altman [10]. The results of this quality control ended the first period of VL testing.

In mid 2007, the internationally supported Lab 2 was re-assessed by an MSF laboratory scientist, and external quality control of VL performance was undertaken with the lab's consent. Ten triplicate specimens, obtained with patient consent and prepared as previously, were sent to laboratories in Europe (Lab 4 and Lab 5) that used COBAS AmpliPrep/COBAS TaqMan HIV-1 Tests (Roche Diagnostics, Basel, Switzerland). This process involved approximately one week of staff time, cost around US$1500 for testing and sending frozen samples with dry ice and triple-packaging by courier, and incurred considerable difficulties in ensuring unbroken cold chain conditions.

Results were compared as for the earlier duplicate testing. After an acceptable outcome of this quality review, VL tests were performed on all patients after at least six months on ART. Specimens were labelled with patient name, ID code and date of collection. Samples were sent in large batches with both electronic and printed lists of these details, all of which were cross checked at the time of specimen storage and again when samples were shipped frozen on ice. Results were provided on these lists, both electronically and printed.

### Ethics approval

After consideration of the MSF Ethics Review Board framework criteria, this paper was assessed as not needing ethics approval. The work described was undertaken as part of routine programme work and analyzed retrospectively. Neither the programme nor individuals have been identified.

## Results

In 2005 and 2006, 178 of 334 (53%) previously ART-naïve patients tested by Lab 1 after six to 12 months of treatment had VLs of less than 1000 copies/mL. However, only 4% showed evidence of immunological failure according to 2006 WHO guidelines [11]. Virological suppression rates of less than 1000 copies/mL in similar MSF programmes in other countries using standardized treatment protocols were higher than 85% [12,13], and in a review of programmes in sub-Saharan Africa, were above 76% [14].

Tolerating variation of up to 1.0 log difference in results (to allow for differences in the test methods used), there was substantial discordance in results of duplicate samples tested by Lab 1 and comparison laboratories (Table 1): results of 13 (62%) samples sent to Lab 2 and 14 (48%) sent to Lab 3 were outside the expected range of difference. Lab 1 refused to provide internal quality control data on future test runs, or to receive and test blinded duplicate samples. Therefore, use of that laboratory was discontinued and VL testing halted. Clinicians were instructed to consider all previous VL test results from Lab 1 invalid (more than 2500 tests on more than 1500 patients at various stages of treatment).

**Table 1:** Results of duplicate sample tests in testing and external quality control laboratories

	Lab 1 vs. Lab 2 2006	Lab 1 vs. Lab 3 2006	Lab 2 vs. Lab 4 vs. Lab 5* 2007
Number of sample sets	21	29	10

Range of log VL results reported	ND-6.1	ND-5.7	ND-4.6

N (%) of duplicate samples with difference in log VL result >1.0	13 (62%)	14 (48%)	1 (10%)

Mean (range) absolute difference in log VL result	1.1 (0.0-2.8)	1.2 (0.0-3.1)	0.2 (0.0-1.2)

Limits of agreement of tests (log VL result)^†^	-4.777 to 0.801	-3.089 to 3.246	-0.796 to 0.848

False negatives: N (%) VL undetectable in testing lab but >1000 copies/mL in QC laboratory [same comparison but >5000 copies/mL]	0	5 (17%) [5 (17%)]	0

False positives: N (%) >1000 copies/mL in testing lab but undetectable in QC laboratory [same comparison but >5000 copies/mL]	6 (29%) [1 (5%)]	8 (28%) [5 (17%)]	0

*Samples were sent in triplicate to Lab 2, Lab 4 and Lab 5. VL = Viral load. QC = Quality control. ND = Not detectable.

†Bland Altman comparison [10]

In 2007, VL testing recommenced in order to review patient status and reassess programme virological suppression rates. Prior to commencement of testing, acceptable external quality control results were obtained for Lab 2 (Table 1) and agreement reached that internal quality control results for each test run would be provided (calibration curve and positive controls).

Results from the first eight test runs (614 samples) performed by Lab 2 in 2007 were released to the programme as valid, but quality control data to verify this were not released concurrently. MSF received VL results from the test runs on average 27 days (range 5-69) after sample receipt by Lab 2, but the internal quality control data were received a further 34 days (range 25-42) later, despite repeated requests. Reasons for VL result delays included laboratory stock shortages caused by delays in importing test kits and a lack of in-country technical support for instrument maintenance and repairs. As a result, more than 300 of the 614 patients tested in the first batch did not receive their VL results at their scheduled follow-up appointment, and clinicians could not use the information for optimum care.

When eventually released, quality control data showed 363 (59%) of the initial 614 samples were invalid because calibration curves or positive controls were outside acceptable limits. These faults were only recognized when data were examined by MSF staff and external international advisers; the expert interpretation was communicated to Lab 2 and the service agreement conditions were further clarified. The official release of results by Lab 2 combined with the delay in receiving quality control data meant that some patients received inaccurate results. Seventy-eight of the 363 patients from these invalid test runs were told that their VLs were greater than 1000 copies/mL, which was the project criteria for enhanced adherence counselling, whereas other results might have been inaccurately low. Lab 2 blamed calibration errors on power failures and an inverter too weak to prevent interruptions when switching from national power to generator. The initial sample volume requested by Lab 2 was inadequate for retesting, and the 363 patients had to be recalled for repeat venepuncture. Of the first 124 of these samples retested, 52 (42%) were in a second invalid run and required further repeat testing.

Sending samples internationally for ongoing quality control was not feasible because of the time, logistics and expense involved. Instead, around 10% of samples after the initial eight test runs were sent in blinded duplicate to Lab 2. Of 46 initial sample pairs in test runs with acceptable quality control, the result for seven (15%) duplicate pairs was greater than 1.0 absolute log difference (maximum 2.7 log).

## Discussion

Although we support the WHO recommendations on routine VL testing in principle, our results question the feasibility of safe implementation in resource-limited settings with current technology in the absence of real-time review of quality control data by an experienced third party. Inaccurate results and quality control delays created the potential for HIV patients to be attributed with "false virological failures". This caused confusion and distress for patients, since ART appeared to be failing despite potentially optimal adherence.

Extra appointments were also needed, which increased time and transport costs. ART might also have been switched to second-line unnecessarily. No regimens were changed on the basis of invalid 2007 results; however, on file review, three changes in 2006 were possibly influenced by VL results later deemed invalid. Second-line treatment carries an increased pill burden and cost, and if true virological failure occurred later, patients might be assumed to have no effective regimens in the absence of third-line ART. Importantly, poor VL performance may have resulted in false negative results, where patients with virological failure were not detected, putting them at risk of severe clinical illness and antiviral resistance.

Retesting patients because of quality control problems was a burden for them and for programme staff, especially as some patients had to be recalled to discuss released, then invalidated, results. This led to apparent frustration, loss of confidence, and inconvenience for patients. Perceived poor VL outcomes led to inaccurate assessments of programme quality, with consequent unnecessary programmatic changes.

There are potential limitations with the quality control analyses with regard to the number of externally tested samples, time of storage, and comparison of VL on duplicate samples using different tests in different laboratories and where subtype was not tested. However, these differing test methods would be expected to give comparable results [15-17]. In view of the large discrepancy between VL results from Lab 1 and immunological outcomes, outcomes achieved with standard MSF protocols in other similar settings, the results of quality control tests, and the subsequent VL results, most of the observed discordance is probably due to poor test performance and lack of appropriate quality control. Additionally, quality issues other than discordant results remain relevant.

We cannot assume that a laboratory will provide accurate results simply because it is supported by a reputable international laboratory and participates in an external quality assurance programme. Sophisticated equipment does not guarantee accurate results [18]. Contractual agreements with laboratories should include remuneration based upon release of valid results with accompanying quality control data. Calibration data should be able to be correctly reviewed and interpreted by staff. Reluctance to release these data (either at all or with delay) should be taken as a "red flag". Lab 2 reported that it had fixed its inconsistent power problem, had the equipment serviced (one reason for test delays), and paid more attention to quality control, yet returned some additional results with invalid quality control. WHO provides guidance on training to improve quality control and quality assurance in the context of complex laboratory testing [19].

Barriers to obtaining reliable laboratory results for complex tests in resource-limited settings include: unpredictable power supplies; shortages of service engineers; expense of reagents in performing repeat test runs; lack of motivation and accountability; and shortages of highly qualified scientific staff [9,18,20]. In addition, although complex equipment may be supplied by funding agencies, quality assurance is not routinely included in their budgets, ongoing access to training and technical support is often not available, and laboratory testing is not seen as a high priority expense [8].

## Conclusions

The resources and expertise needed to ensure accuracy make it unlikely that all programmes will be able to achieve sufficient confidence in VL results. Worryingly, many programmes are already using VL technology routinely in settings where it is difficult to ensure the quality of laboratory services [17]. Our results indicate a crucial need for these programmes to review the accuracy of VL results. More adapted, reliable, cheap and point-of-care methods of VL testing are urgently required for resource-limited settings [21].

Our experience of the difficulties encountered in this programme and other programmes in eastern Europe, Asia and other countries in sub-Saharan Africa led to development of a guide for assessing a laboratory's suitability for VL (or other) testing (See additional file 1: Checklist). It aims to minimize the likelihood of obtaining inaccurate results, focusing on the analytic phase of testing, and could help ensure quality results.

Although the quality of VL testing may be reliable in many resource-limited settings, the need to ensure that this is true applies to all laboratories in these settings. Despite cost and logistical issues, blinded duplicate samples should be sent to the testing laboratory, with investigation triggered by a simple rule violation of any identical samples being outside the expected range of difference.

## Competing interests

PRH declares grant funding and/or consulting relationships with Virco, Viiv, Quest, Pfizer, Abbott and Merck. The other authors declare that they have no competing interests.

## Authors' contributions

JG, PDC, CM, DK and DOB contributed to the study conception and design. JG, DK and DOB collected and analyzed data. JG and DOB wrote the first draft of the paper. DK and PDC contributed to the writing of the paper. CM, SJ and PRH reviewed the paper. All authors read and approved the final manuscript.

## Supplementary Material

Additional file 1 Checklist Click here for file
