# Supplementary material for: Viral load testing in a resource-limited setting: quality control is critical
Source: J Int AIDS Soc. 2011 May 12;14:23. doi: 10.1186/1758-2652-14-23 (PMC3116452; doi:10.1186/1758-2652-14-23)
Supplement: Additional file 1 — Checklist [file 1758-2652-14-23-S1.DOC]

## Checklist for considering an external laboratory for viral load testing

1. Document programme requirements and capacity:
   - Number of tests expected per week and in total
   - Required time frame for receiving results
   - Capacity to store specimens safely and properly:
   - Trained staff; infectious specimens separate from main cold chain; freezer with prioritized constant power and reliable temperature less than –20°C with routine monitoring; storage limited to <4 weeks (-80°C required for longer storage)
   - Capacity to store duplicate samples (–80°C needed if storage for >4 weeks)
   - Capacity to transport specimens to the testing laboratory in appropriate conditions:
   - Ice blocks if transport <2 hours, otherwise dry ice
   - If using a courier/airline, can they accept consignments containing dry ice and track and guarantee cold chain?
   - If the testing laboratory is out-of-country, do you need export/import permits?
   - Ability to obtain external advice on technical issues within a reasonable time frame
   - One (preferably two) key person for contact with the testing laboratory, specimen/results management, and interpretation of quality control data
   - Medical staff must understand the value and limitations of the test in their context.
2. Perform an initial quality assessment of potential laboratory (a combination of the following is preferable):
   - It is a recognised reference laboratory
   - It is directly supervised by a recognized reference laboratory
   - It is accredited in a national laboratory accreditation programme
   - It provides results in an external quality control programme
   - An expert of your choice inspects and approves the laboratory. Criteria may include:
   - Specific areas for reagent preparation, sample preparation and sample analysis
   - Steps to avoid cross contamination (workflow and decontamination of work area)
   - Access to water of appropriate quality (reagent grade and preferably PCR grade)
   - Guidelines for decontamination of equipment in place
   - Standard operating procedures in use
   - Review of personnel training manual, equipment validation data, laboratory equipment maintenance programme (daily, weekly and monthly maintenance).
   - Backup power for laboratory freezers and refrigerators, and temperature monitoring guidelines.
3. Assess laboratory capacity:
   - Regularly performs the required test using a method that is valid in your setting (e.g., appropriate for relevant subtypes)
   - Can meet requirements for specimen number and testing/reporting time frame
   - Has a stock management system that can assure supplies (e.g., import/purchase restrictions will not result in stock shortages)
   - Can have test equipment serviced, repaired and calibrated within reasonable time frame
   - Has a reliable power supply (sufficient backup/stabilizer to prevent ANY interruptions or fluctuations during a test run)
   - Has at least one (preferably two) technician(s) trained and experienced with the test who will not be routinely absent
   - Check extent of training/experience with test procedure, understanding of limitations of test and interpretation of results and potential causes of error
   - Check availability of training as refresher or for replacement staff
   - Has more than one contact person to provide results and advise of delays
   - Willing to be open, in good faith, about unexpected issues or delays
   - Willing to accept regular blinded quality control samples (sent at your cost as parallel blinds)
   - Willing to give quality control data for test runs on your samples
   - Committed to quality control and agrees on conditions for releasing test runs or rejecting and repeating a run (at their cost)
   - Willing to store reserve samples at –80°C sufficient to enable retesting until test run passes quality control
   - Ensure volume needed for two tests known and achievable.
4. Initial quality control assessment:
   - Send ≥10 samples for testing in parallel by a recognized reference laboratory that uses a relevant test method. The results should be within the precision of the methodologies as specified by the instrument manufacturers.
5. Prepare a memorandum of understanding to cover the above issues (especially quality control responsibilities):
   - Keep perspective that the aim is good patient care, not policing the laboratory.
6. Ongoing quality control assessment:
   - Appoint a trained staff member to approve test lab calibration data before results are made available to clinical staff
   - Routinely include blinded duplicate samples in batches sent to the test laboratory. Any results outside the expected range of difference (0.9 log is generous for VL) between identical duplicates should trigger an investigation to determine the cause.
